# Entrapment Neuropathies in the Upper and Lower Limbs: Anatomy and MRI Features

**DOI:** 10.1155/2012/230679

**Published:** 2012-10-17

**Authors:** Qian Dong, Jon A. Jacobson, David A. Jamadar, Girish Gandikota, Catherine Brandon, Yoav Morag, David P. Fessell, Sung-Moon Kim

**Affiliations:** Division of Musculoskeletal Radiology, Department of Radiology, University of Michigan Health System, 1500 East Medical Center Drive, TC 2910R, Ann Arbor, MI 48109-5326, USA

## Abstract

Peripheral nerve entrapment occurs at specific anatomic locations. Familiarity with the anatomy and the magnetic resonance imaging (MRI) features of nerve entrapment syndromes is important for accurate diagnosis and early treatment of entrapment neuropathies. The purpose of this paper is to illustrate the normal anatomy of peripheral nerves in the upper and lower limbs and to review the MRI features of common disorders affecting the peripheral nerves, both compressive/entrapment and noncompressive, involving the suprascapular nerve, the axillary nerve, the radial nerve, the ulnar nerve, and the median verve in the upper limb and the sciatic nerve, the common peroneal nerve, the tibial nerve, and the interdigital nerves in the lower limb.

## 1. Introduction

Peripheral neuropathies are relatively common clinical disorders, which may be classified, according to cause, into compressive or entrapment and noncompressive neuropathies [1]. Although nerves may be injured anywhere along their course, peripheral nerve compression or entrapment occurs more at specific locations, such as sites where a nerve courses through fibroosseous or fibromuscular tunnels or penetrates muscles [2, 3]. Typically, the diagnosis has been based mainly on the combination of clinical history, physical examination, and electrodiagnostic studies. However, such clinical evaluations may provide insufficient information in making an accurate diagnosis, and imaging is being used often to confirm diagnoses.

Magnetic resonance imaging (MRI) and high-resolution ultrasonography (US), as noninvasive techniques, provide valuable spatial information in making important diagnostic distinctions that cannot be readily accomplished by using other existing methods [2, 4, 5]. While both allow direct anatomic visualization of a nerve, identification of the cause, and location of primary abnormalities, MRI has the ability to demonstrate intrinsic signal abnormalities within the nerve itself and is considered superior in delineating the associated indirect signs related to muscle denervation [2, 4].

The purpose of this paper is to illustrate the anatomical course of peripheral nerves and review a broad spectrum of common peripheral neuropathies, both compressive/entrapment and noncompressive, involving the suprascapular, axillary, radial, ulnar, and median nerves in the upper limb, and the sciatic, common peroneal, tibial, and the interdigital nerves in the lower limb.

## 2. MRI Features

 The signal intensity of a normal nerve on MRI is of intermediate to low on T1-weighted sequences becoming slightly higher on T2-weighted and other fluid-sensitive sequences [3, 4]. Enlargement with apparent increase in T2 signal is considered an abnormal MRI appearance [3]. In addition, a hyperintense signal of the denervated muscle is usually identified when entrapment is acute, and fatty infiltration and muscle atrophy are the signs of chronic neuropathy in longstanding cases [2–4].

Peripheral nerve sheath tumors (PNSTs) appear as a well-defined mass continuous with a peripheral nerve. Such continuity with the nerve produces the “split fat sign,” where the normal fat around the peripheral nerve is split around the neuroma [6]. Benign PNSTs usually show intermediate signal on T1-weighted images, while on fluid sensitive sequences the tumor shows high signal. The finding of central low signal surrounded by high signal on fluid sensitive sequences (a target appearance) suggests that the PNST is benign [6]. Schwannomas tend to be eccentric to the nerve trunk in comparison with neurofibromas, although this can be variable. Malignant PNSTs do not display the target appearance and are often heterogeneous with necrosis [6]. There is variable contrast enhancement at MR imaging in both benign and malignant PNSTs, with the pattern of enhancement commonly either heterogeneous and diffuse or peripheral. Generally, more contrast enhancement is apparent in malignant PNSTs [6]. 

## 3. Upper Limb

### 3.1. Suprascapular Nerve

**Figure 1:**
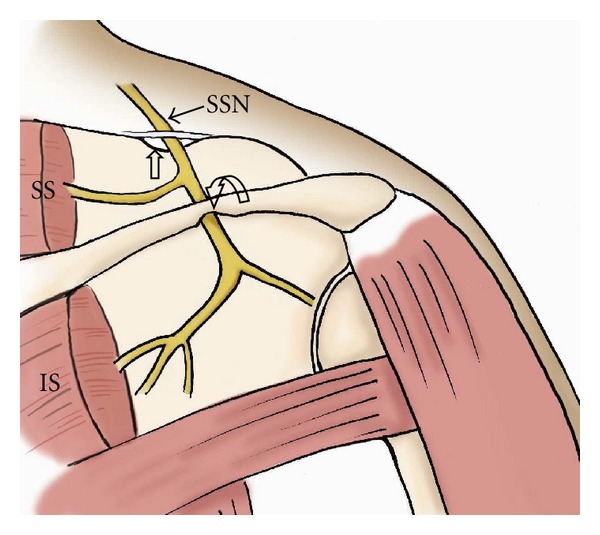
The drawing shows anatomy of the suprascapular nerve from the posterior view. Note the nerve courses through the suprascapular notch (open arrow) and spinoglenoid notch (curved arrow). SSN: suprascapular nerve, SS: supraspinatus muscle, IS: infraspinatus muscle.

The suprascapular nerve enters the supraspinatus fossa through the suprascapular notch, which is a fibroosseous tunnel bridged by the transverse scapular ligament. In the suprascapular fossa, the suprascapular nerve gives off two branches to the supraspinatus muscle and the superior aspect of the shoulder joint. The remaining portion of the nerve travels around the lateral margin of the scapular spine, through the spinoglenoid notch, and into the infraspinatus fossa to supply the infraspinatus muscle and posterior aspect of the glenohumeral joint (Figure 1) [7, 8].

**Figure 2:**
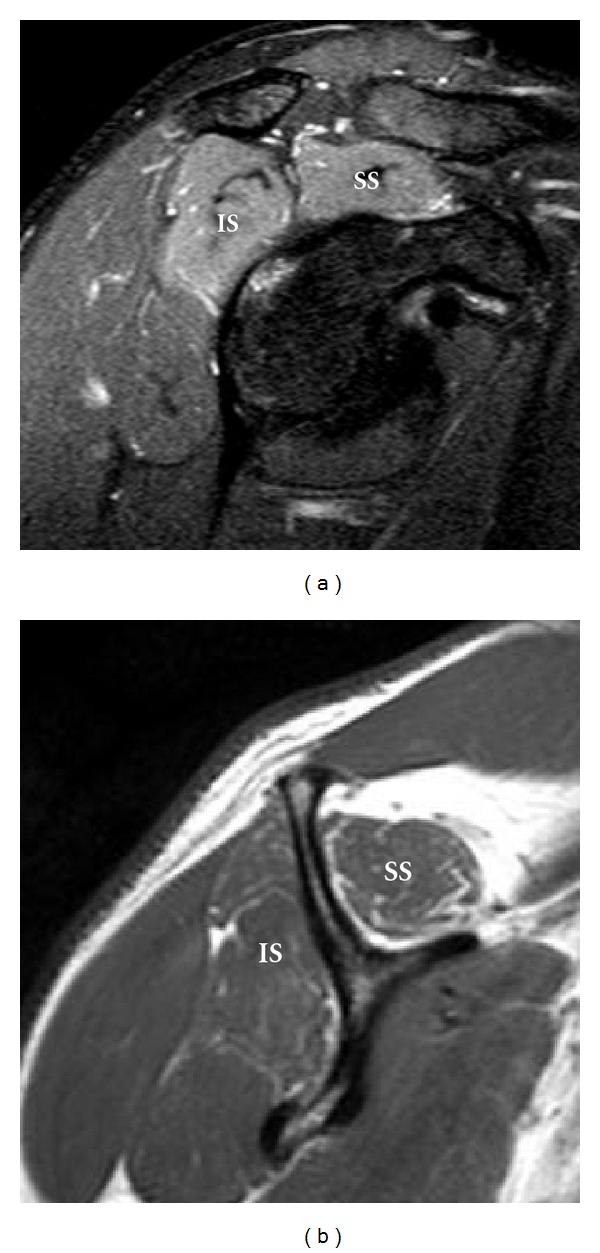
A 44-year-old patient after a swimming accident with clinical and EMG evidence of right supraspinatus and infraspinatus muscle denervation at the suprascapular notch. High signal intensity and mild muscle atrophy with fatty infiltration involving the supraspinatus (SS) and infraspinatus (IS) muscles are demonstrated on sagittal oblique T2 fat-saturated (a) and T1-weighted (b) images.

**Figure 3:**
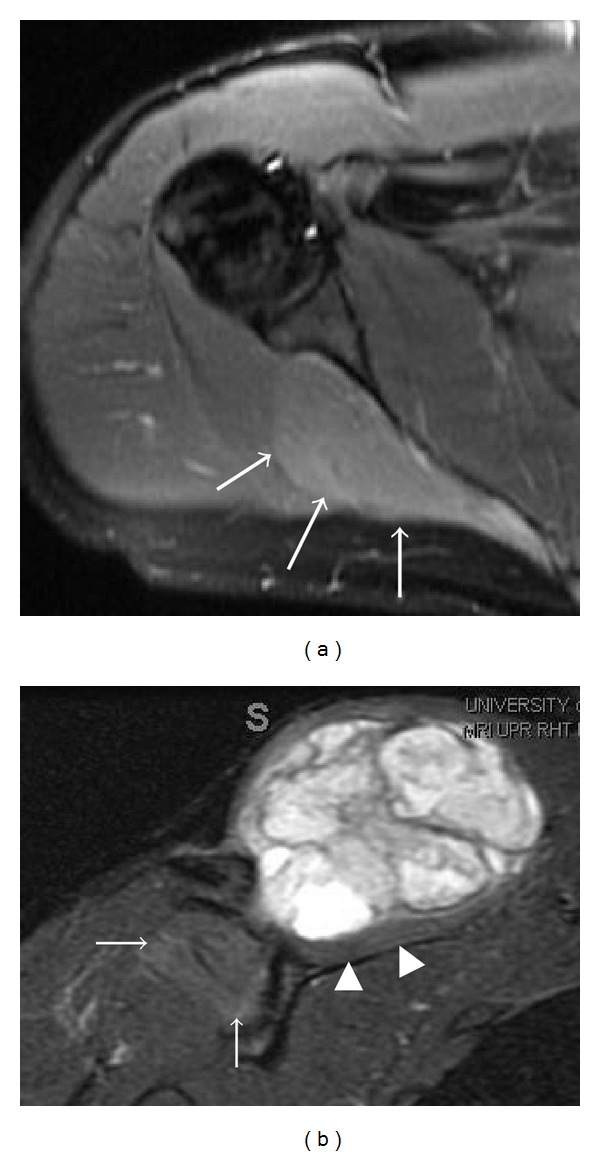
A high-grade sarcoma in a 28-year-old patient has MRI appearances indicating a right suprascapular nerve entrapment by the tumor at the spinoglenoid notch. Isolated increased signal intensity in the infraspinatus muscle (arrows) on axial (a) T2 fat-saturated image, with mild enhancement on coronal (b) postcontrast T1 fat-saturated image. The heterogeneous enhancing tumor produces mass effect on the supraspinatus muscle (arrowheads), which has normal signal intensity.

Suprascapular nerve compression or entrapment, known as suprascapular nerve syndrome, can occur as a result of trauma, an anomalous or thickened transverse scapular ligament, or extrinsic compression by a space-occupying lesion [7, 8], commonly a ganglia cyst or soft tissue tumor. Compression or entrapment at the suprascapular notch leads to supraspinatus and infraspinatus muscle denervation (Figure 2), whereas more distal entrapment at the spinoglenoid notch may present with isolated involvement of the infraspinatus muscle (Figure 3). Patients may present with poorly localized pain and discomfort at the back of the shoulder or the upper back, as well as weakness when raising the arm.

MR imaging may allow early recognition of suprascapular nerve entrapment, by detecting clinically unsuspected masses, as well as indirect signs of nerve denervation in suprascapular nerve syndrome [7, 8].

### 3.2. Axillary Nerve

The axillary nerve enters the quadrilateral space with the circumflex humeral artery. It supplies the teres minor and deltoid muscles and the overlying skin of the shoulder.

**Figure 4:**
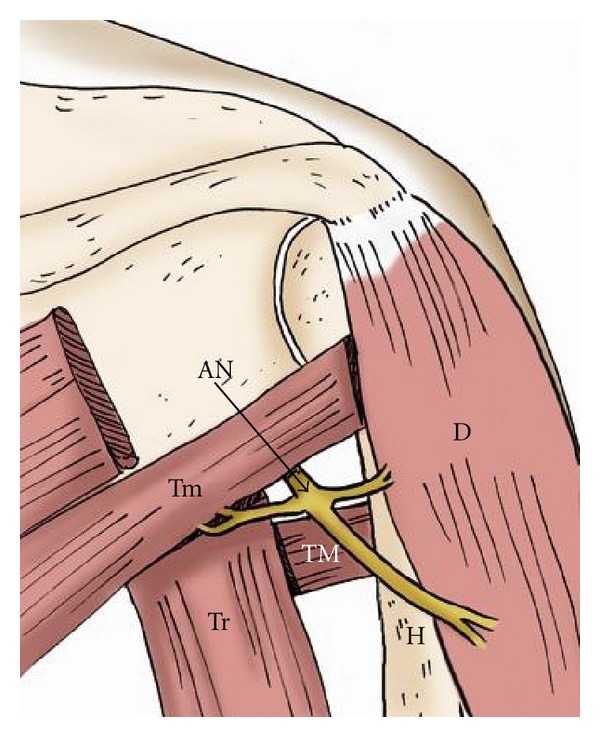
The drawing shows the axillary nerve within the quadrilateral space from a posterior view. AN: axillary nerve, Tm: teres minor muscle, Tr: long head of the triceps, TM: teres major muscle, H: humerus, D: deltoid muscle.

The quadrilateral space is bounded by the teres minor, teres major, long head of the triceps, and the neck of the humerus (Figure 4).

Quadrilateral space syndrome is an uncommon condition in which the posterior circumflex humeral artery and the axillary nerve are compressed within the quadrilateral space. Other than dynamic compression due to abduction and external rotation of the shoulder joint, abnormal fibrous bands and hypertrophy of the adjacent musculature as well as space-occupying lesions are other causes [9, 10]. Clinical manifestations include poorly localized shoulder pain and paresthesias in the affected arm in a nondermatomal distribution. The diagnosis can be difficult since clinical symptoms may be confused with rotator cuff pathology or other shoulder joint-related abnormalities [9].

**Figure 5:**
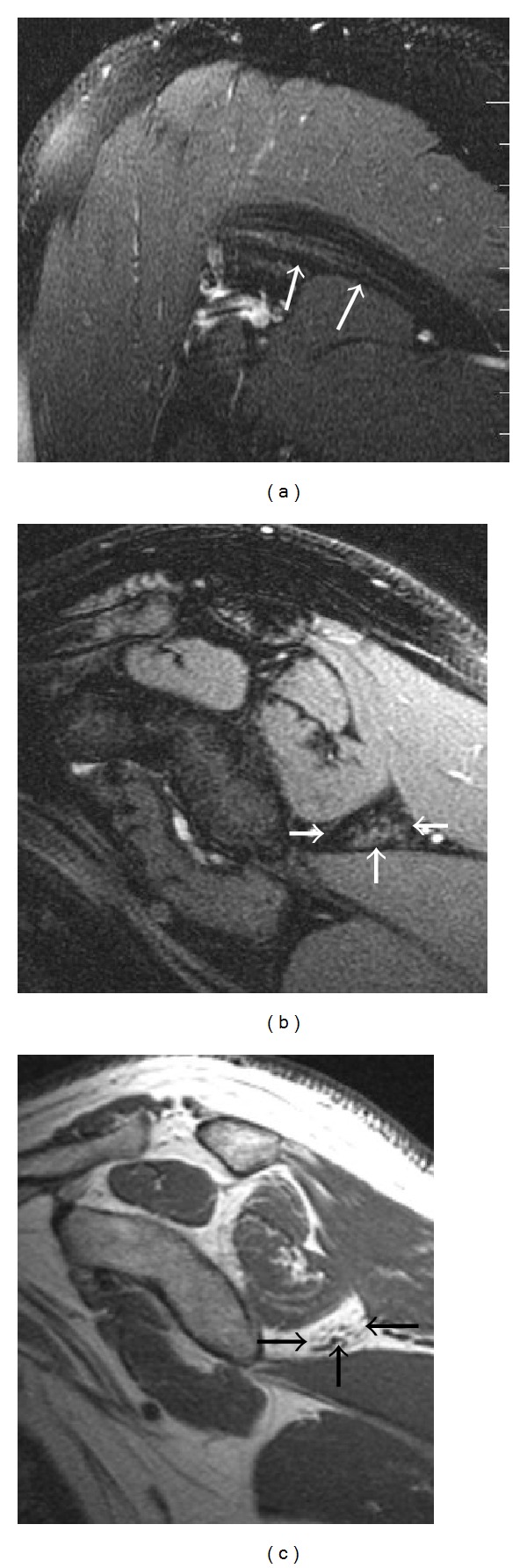
A 46-year-old patient with right shoulder pain and clinical and EMG evidence of quadrilateral space syndrome. Oblique coronal (a), oblique sagittal (b) T2 fat-saturated, and oblique sagittal T1-weighted (c) images demonstrate severe fatty atrophy of the teres minor muscle (arrows).

The axillary neurovascular bundle within the quadrilateral space can be defined on oblique sagittal T1-weighted images. In quadrilateral space syndrome, MRI may show signal alteration or atrophy of the teres minor muscle with or without involvement of the deltoid muscle (Figure 5) [9]. In addition, MRI has superior ability in delineating any space-occupying lesions. It is important to realize that isolated atrophy or abnormal signal in the teres minor muscle may occur in asymptomatic patients or patients with other shoulder abnormalities [9]. Therefore, care must be taken to assess the entire shoulder with clinical correlation when such imaging findings are present on MRI.

### 3.3. Radial Nerve

**Figure 6:**
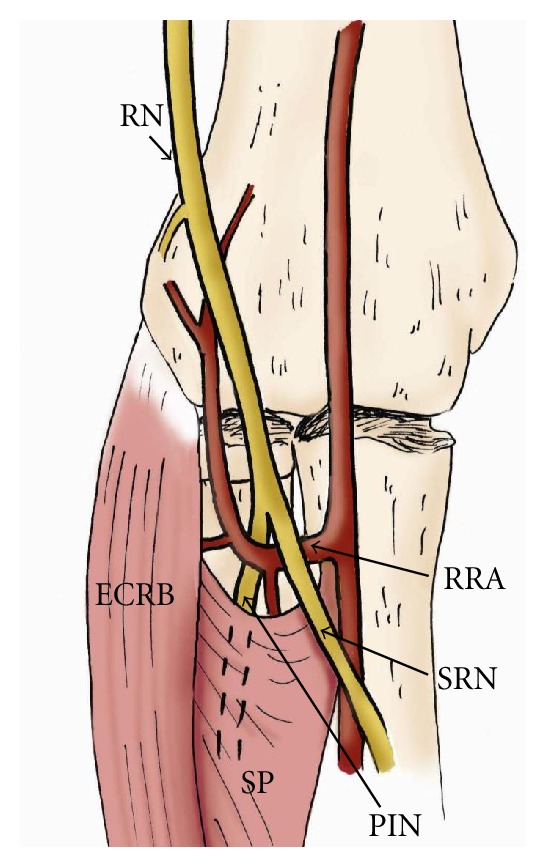
The drawing provides an anterior view of the course of the radial nerve at the elbow. Posterior interosseous nerve (PIN) entrapment may occur due to prominent radial recurrent artery (RRA), medial edge of the extensor carpi radialis brevis (ECRB), and proximal edge of the supinator muscle (SP) (arcade of Frohse). RN: radial nerve, SRN: superficial radial nerve.

While traveling along the posterior upper arm through the triceps muscle, the radial nerve runs in the spiral groove which is located in the lateral and posterior aspect of the mid humeral diaphysis. At the level of the elbow joint, the radial nerve divides into the posterior interosseous nerve (PIN) (deep branch) and the superficial branch of the radial nerve. Close to its origin, the PIN is crossed by the lateral branches of the recurrent radial artery and vein, the so-called leash of Henry. The PIN then descends deep in relation to the proximal edge of the superficial layer of the supinator muscle, which is known as the arcade of Frohse (Figure 6). The superficial branch of the radial nerve courses alongside the radial artery and then distally courses lateral over the first extensor wrist compartment [9].

The radial nerve is predisposed to injury and entrapment at several locations along its course, which include the radial nerve in the spiral groove of the humerus (spiral groove syndrome) above the elbow joint, where the PIN travels through the radial tunnel, and the superficial branch of the radial nerve where it crosses over the first dorsal wrist compartment (Wartenberg's syndrome).

**Figure 7:**
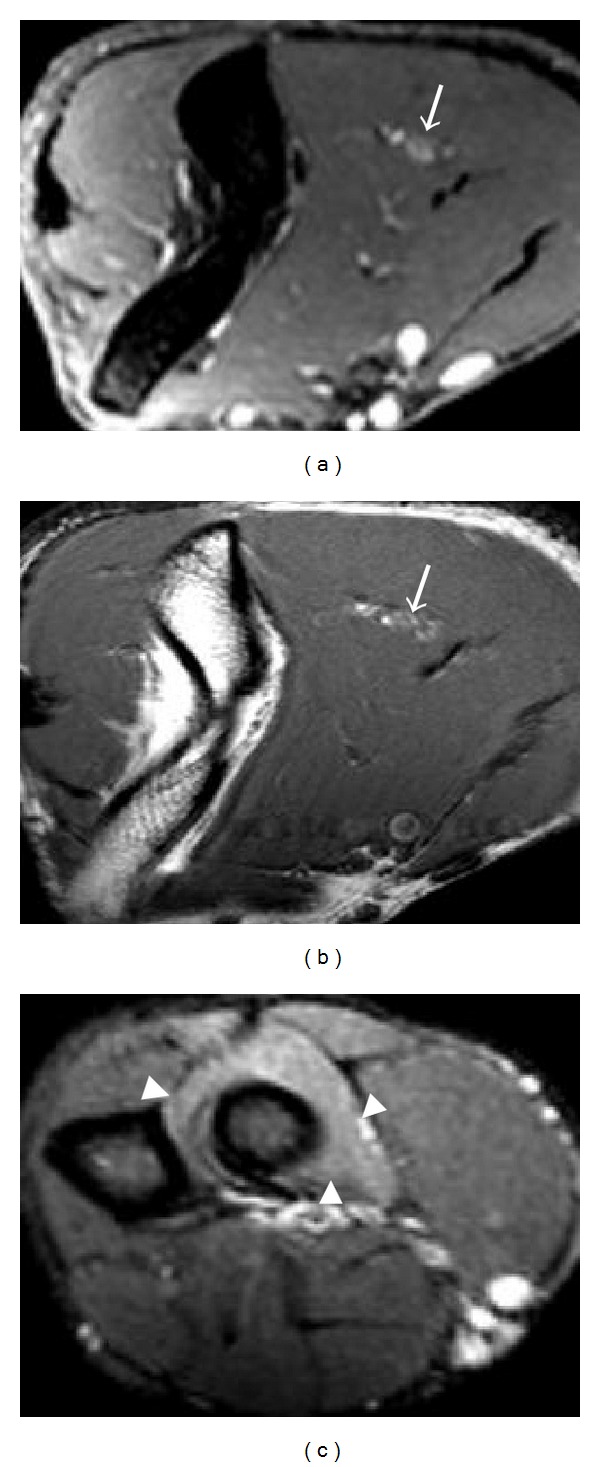
An 18-year-old patient with clinical and EMG evidence of PIN entrapment. Axial T2 fat-saturated (a) and T1-weighted (b) images at the level of right distal humerus show thickening and high T2 signal of the radial nerve (arrows). Supinator muscle edema (arrowheads) is present (c), axial T2 fat-saturated image.

Compression or entrapment of the PIN in the radial tunnel may yield two different clinical presentations: posterior interosseous nerve syndrome and radial tunnel syndrome. The radial tunnel is a musculoaponeurotic furrow or space extending from the lateral epicondyle of the humerus to the distal edge of the supinator muscle [11]. In patients with posterior interosseous nerve syndrome, the clinical presentation includes motor deficits of the extensor muscle group without significant sensory loss. The most common site of nerve compression of PIN within the radial tunnel is posterior interosseous nerve syndrome at the arcade of Frohse. Other structures can potentially cause compression or entrapment including the medial edges of the extensor carpi radialis brevis, fibrous bands at the radial head, and the leash of Henry [12] (Figure 6). Patients with radial tunnel syndrome, on the other hand, typically present with pain over the proximal lateral forearm [12, 13], which can be caused by acute trauma, masses, and compression from adjacent structures. MR imaging features range from direct visualization of nerve thickening with increased T2 signal and muscle signal alterations (Figure 7) to the detection of compressive lesions or abnormal structures, which may cause compression or entrapment as mentioned above.

Entrapment of the superficial branch of the radial nerve at the level of distal wrist is called Wartenberg's syndrome [5]. The superficial location of the superficial branch of the radial nerve predisposes it to injury and may result from fixation of a distal radius fracture, penetrating injury, and iatrogenic injury related to vein cannulation or adjacent tendon sheath injection [5].

### 3.4. Ulnar Nerve

**Figure 8:**
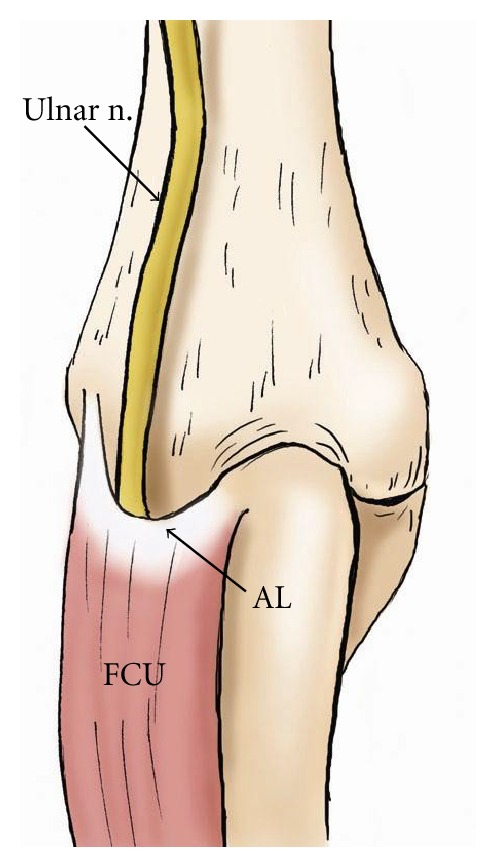
The drawing demonstrates the course of the ulnar nerve from posterior view at the elbow. Note the nerve travels deep to the flexor carpi ulnaris muscle (FCU) beneath the arcuate ligament (AL).

The ulnar nerve enters the cubital tunnel, a fibroosseous canal, at the level of the medial epicondyle of the elbow. The roof of the tunnel is formed by a fascial band between the olecranon process and the medial epicondyle known as the cubital tunnel retinaculum [14]. The nerve then passes beneath the arcuate ligament which is an aponeurosis between the humeral and ulnar heads of the flexor carpi ulnaris muscle (Figure 8). The cubital tunnel retinaculum and arcuate ligament typically blend with each other. After passing through the anterior compartment of the forearm, the ulnar nerve, artery, and vein enter the wrist through Guyon's canal (Figure 10), which is a fibroosseous tunnel between the pisiform and the hook of the hamate, with the roof formed proximally by the palmar carpal ligament and distally by the short palmar muscle, and the floor formed by the flexor retinaculum. Within the canal, the ulnar nerve divides into the superficial sensory and deep motor branches [14].

Compressive or entrapped ulnar nerve neuropathies include cubital tunnel syndrome and Guyon's canal syndrome.

**Figure 9:**
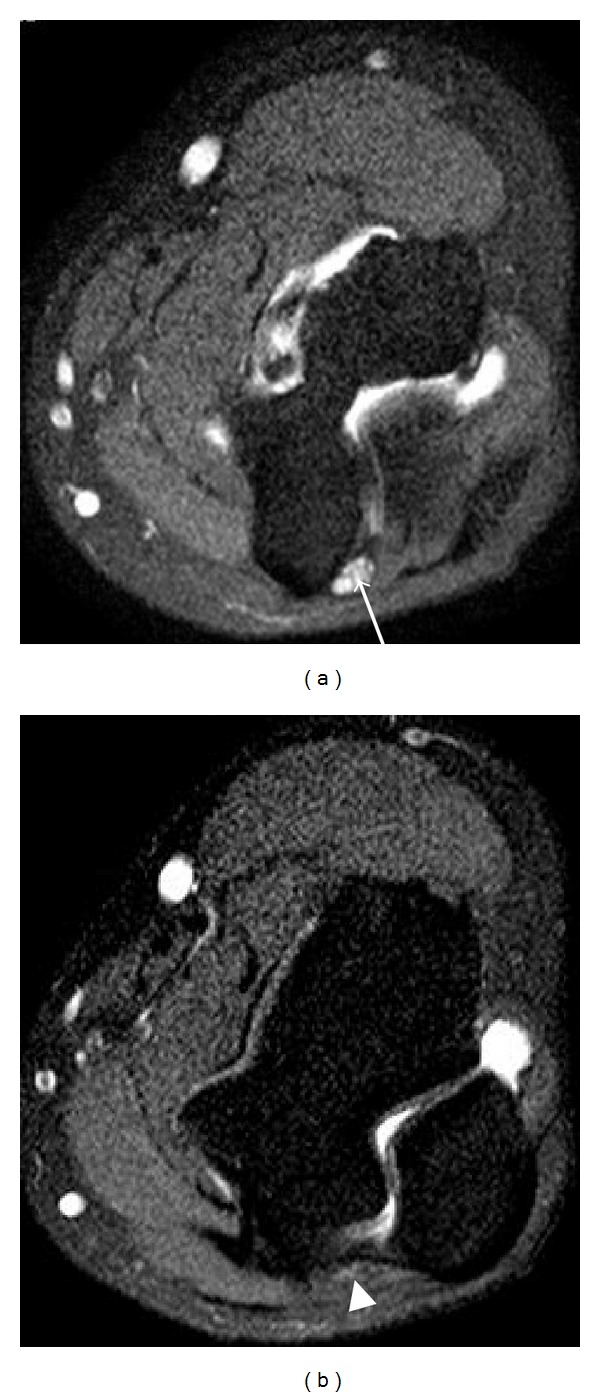
A 17-year-old patient suffered right cubital tunnel syndrome. MR arthrogram of the elbow was performed to evaluate for loose body. Axial T2-weighted with fat-saturated (a, b) images reveal swollen and edematous ulnar nerve (arrow) at the level of proximal cubital tunnel, and normal size distally (arrowhead).

**Figure 10:**
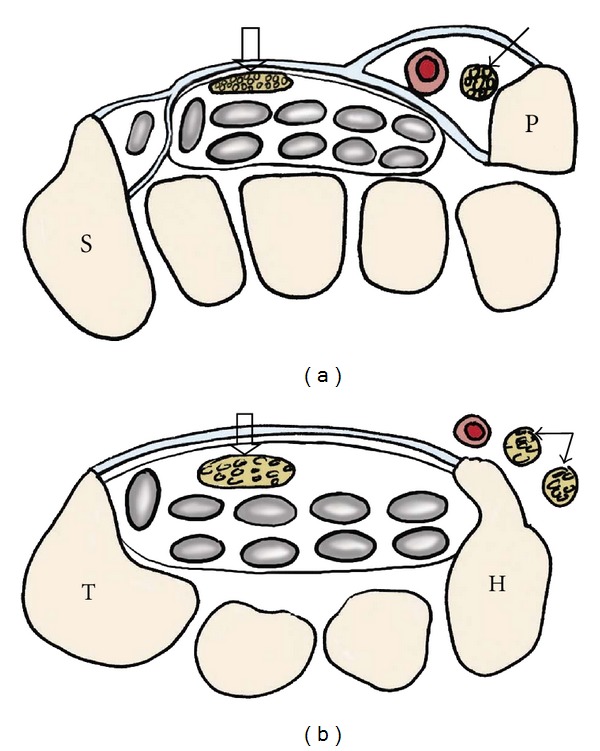
Drawings of the carpal tunnel and Guyon's canal at the levels of pisiform (a) and hamate (b). The median nerve (open arrows) passes through the carpal tunnel under the flexor retinaculum lying superficial to the flexor digitorum superficialis tendons. Guyon's canal contains the ulnar nerve (arrows) and ulnar artery with the floor formed by the flexor retinaculum. The ulnar nerve divides into the superficial sensory and deep motor branches at the level of the hamate. P: pisiform, S: scaphoid, T: trapezium, H: hamate.

Cubital tunnel syndrome is the second most common peripheral neuropathy of the upper extremity. It may be caused by abnormal fascial bands, subluxation, or dislocation of the ulnar nerve over the medial epicondyle, trauma, or direct compression by soft tissue masses. Clinical symptoms include a sensory abnormality of the ulnar hand and weakness of the flexor carpi muscle group of the 4th and 5th fingers. MRI findings of cubital tunnel syndrome are enlargement and hyperintense signal just proximal to the cubital tunnel, and a caliber change with flattening distally (Figure 9). Mild enlargement and edema of the ulnar nerve at the medial epicondyle can be seen in asymptomatic individuals on MRI [15]. Careful assessment of the flexor muscle group for signal abnormalities and correlation with the patient's symptoms are important.

**Figure 11:**
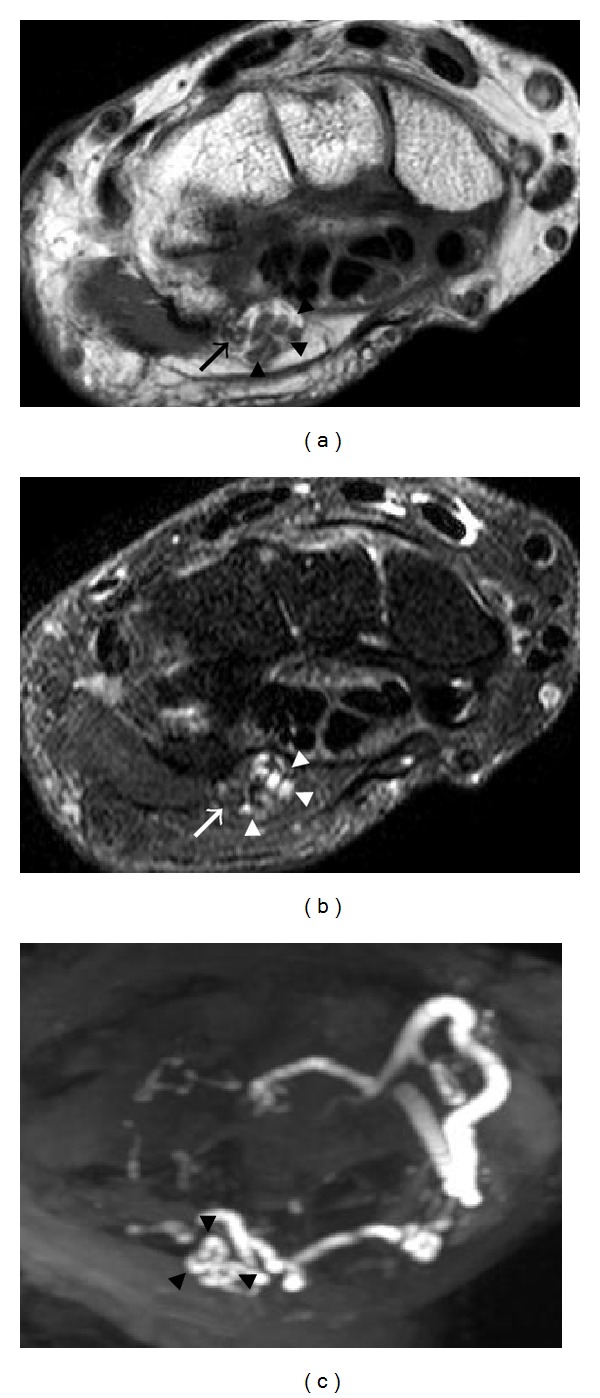
A 73-year-old patient with clinical evidence of right ulnar nerve compression at wrist. Axial T1-weighted (a), T2-weighted with fat saturation (b), and axial SENSE MIPs (c) identify the ulnar nerve (arrows) in a crowded Guyon's canal compressed by a tortuous ulnar artery (arrowheads).

The causes of Guyon's canal syndrome include space-occupying lesions, trauma, anomalous muscles, and ulnar artery aneurysms. Entrapment at Guyon's canal can be associated with either motor or sensory findings, depending on whether the nerve compression involves the ulnar nerve prior to its bifurcation to the superficial (sensory) and deep (motor) branches or if the compression is limited to one of the branches [5]. MR imaging is useful in depicting the ulnar nerve in Guyon's canal demonstrating the etiology of entrapment with additional information of muscle denervation (Figure 11), if present.

### 3.5. Median Nerve

**Figure 12:**
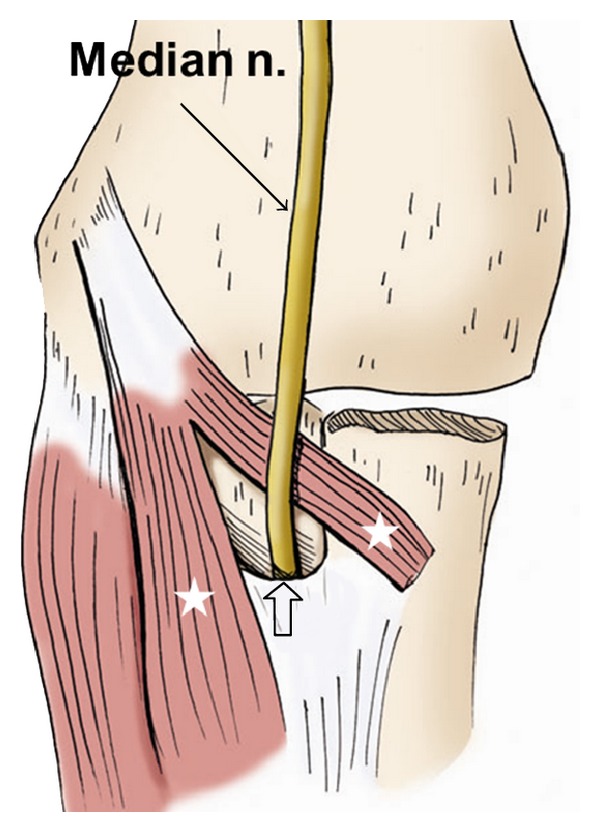
The drawing of the median nerve shows that it courses along the anterior elbow, through the two heads of the pronator teres muscle (stars), and into the forearm beneath the edge of the fibrous arch of the flexor digitorum sublimis (open arrow).

The median nerve enters the forearm between the humeral and ulnar heads of the pronator teres muscle, where the anterior interosseous branch is given off (Figure 12). The nerve then continues distally in the forearm sandwiched between flexor digitorum profundus and flexor digitorum superficialis muscles. It then passes through the carpal tunnel under the flexor retinaculum, lying superficial to the flexor digitorum superficialis tendons. The carpal tunnel is formed by the carpal bones (floor), the transverse carpal ligament (roof), the scaphoid and trapezium (radial side), and the pisiform and hook of the hamate (ulnar side) (Figure 9). The median nerve subdivides into digital and muscular branches as it exits the carpal tunnel.

Median nerve compression or entrapment neuropathies include pronator syndrome, anterior interosseous syndrome, and carpal tunnel syndrome.

Pronator syndrome is relatively rare and is produced by compression or entrapment of the median nerve between the ulnar and humeral heads of the pronator teres muscle. The causes of pronator syndrome may relate to trauma, congenital abnormalities, or pronator teres hypertrophy [1]. Clinical findings include pain and numbness of the volar aspect of the elbow, forearm, and wrist without muscle weakness. A pattern of muscle denervation that is high signal edema on fluid-sensitive sequences is the main MR finding unless there is a mass or hematoma as the secondary cause [1].

Anterior interosseous nerve syndrome (Kilon-Nevin syndrome) is caused by entrapment of the anterior interosseous nerve in the proximal forearm. The causes of anterior interosseous nerve syndrome are similar to those described in pronator syndrome, including direct nerve trauma or compression from a hematoma or mass. Patients may present clinically with pain and muscle weakness in the volar forearm [1]. MR imaging is helpful to show signal intensity changes related to muscle denervation, typically involving the flexor digitorum profundus, flexor pollicis longus, and pronator quadratus muscles [1].

**Figure 13:**
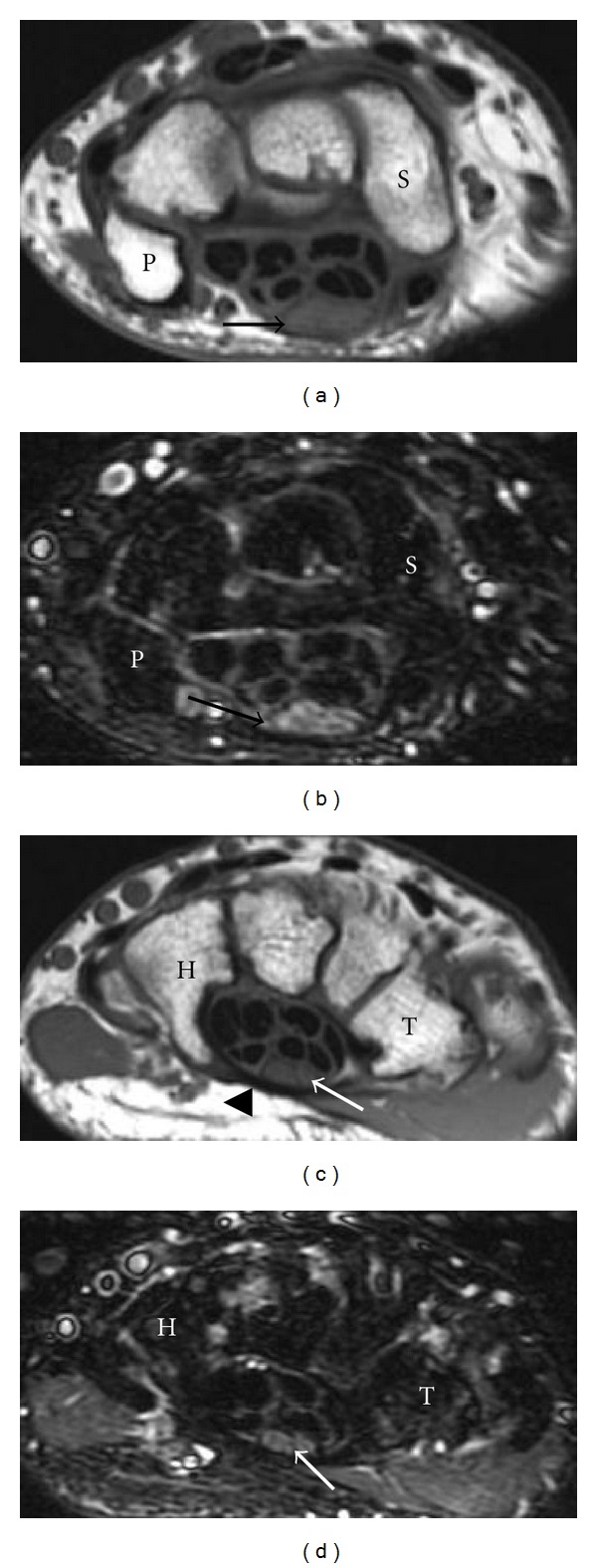
A 48-year-old patient with surgically proven right carpal tunnel syndrome. There is thickening and increased signal intensity of the median nerve (black arrows) at the level of pisiform (P) and bowing of the flexor retinaculum (arrowhead) with a flattened median nerve (white arrows) at the level of hamate (H). (a) and (c) are T1-weighted images, and (b) and (d) are T2-weighted images with fat saturation.

Carpal tunnel syndrome is by far the most common cause of compressive/entrapment neuropathy. Although many cases are idiopathic, it may result from a wide variety of etiologies, including repetitive trauma, conditions related to metabolic and hormonal changes, and ganglion cysts [1, 16]. Patients with carpal tunnel syndrome may experience burning wrist pain, and paresthesia or numbness in the 1st through 3rd fingers, and the radial aspect of the 4th finger. The MR features of carpal tunnel syndrome have been well described, and axial views are the most useful images to demonstrate carpal tunnel syndrome changes. Enlargement of the median nerve is often observed within the proximal carpal tunnel (at the level of the pisiform) in patients with this syndrome. More distally over the carpal tunnel at the level of the hamate, the nerve becomes flattened with bowing of the flexor retinaculum [1, 16] (Figure 13), and a hyperintense signal of the nerve on T2-weighted or STIR imaging is often observed. Although the sensitivity and specificity of the MR findings for carpal tunnel syndrome are low (sensitivity, 23%–96%; specificity, 39%–87%), MR imaging is useful in detecting a space-occupying lesion, inflammatory arthritis, or a congenital anomaly as the cause of carpal tunnel syndrome [1].

## 4. Lower Limb

### 4.1. Sciatic Nerve

**Figure 14:**
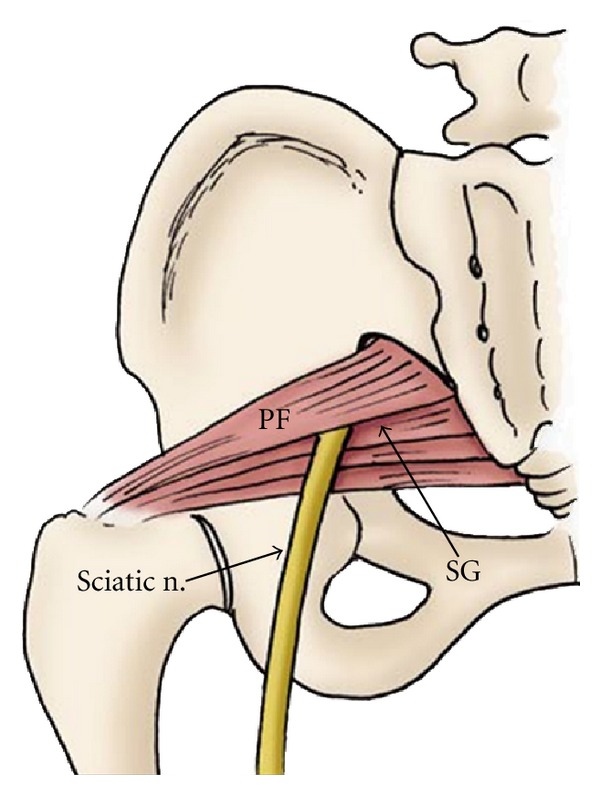
The drawing shows the proximal course the sciatic nerve passing inferior to the piriformis muscle (PS). SG: superior gemellus muscle.

The sciatic nerve originates from the upper division of the sacral plexus and typically leaves the pelvis through the greater sciatic foramen at the inferior border of the piriformis muscle (Figure 14). The sciatic nerve then divides into the tibial and common peroneal nerves just above the knee.

**Figure 15:**
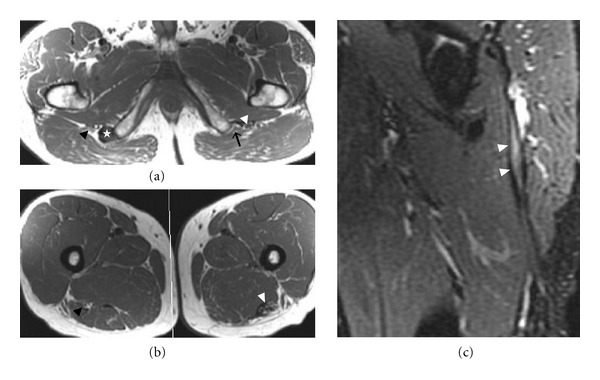
Extensive tear of the left hamstring muscle origin with sciatic nerve scarring in a 54-year-old patient after a water skiing injury. Axial T1-weighted images (a) and (b) identify a tear of left hamstring at the level of origin (black arrow in (a)). There is thickening and abnormal signal of the adjacent sciatic nerve (white arrowheads) extending distally (b), consistent with secondary entrapment from scarring. This finding is also demonstrated on a sagittal fat-saturated T2-weighted image (c). On the right side, the right sciatic nerve (black arrowheads) has normal caliber and signal intensity, and an intact hamstring muscle origin is present (star).

**Figure 16:**
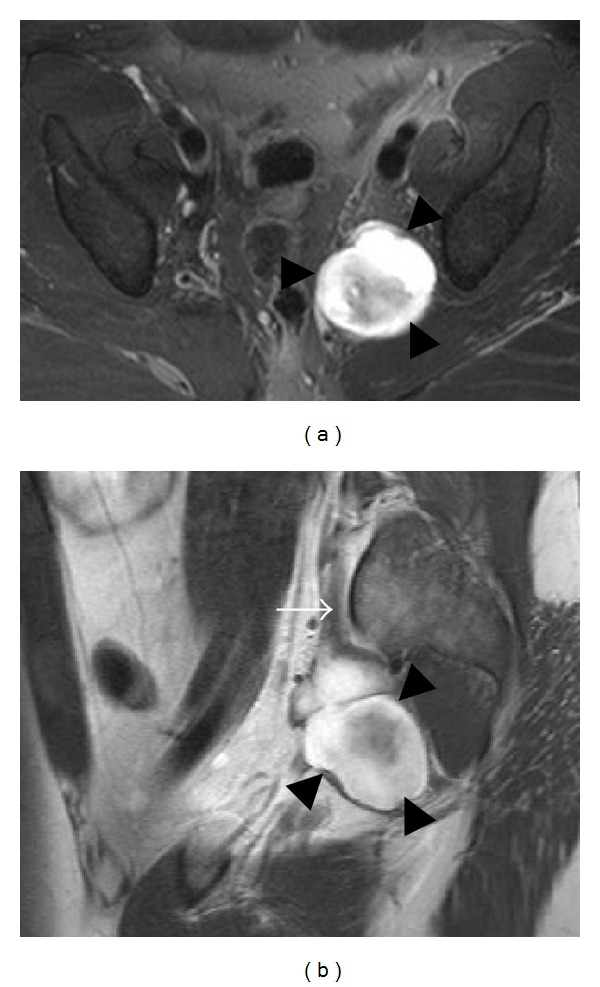
Surgically proven neurofibroma of the left sciatic nerve in a 33-year-old patient. Axial T2-weighted fat-saturated (a) and sagittal T1-weighted postcontrast (b) images show a lobulated enhancing mass (arrowheads) with isointense T1 (not shown) and high T2 signal originating from the left sciatic nerve. Proximally, the left S1 nerve root is thickened (white arrow). Note the target appearance of the neurofibroma.

Sciatic nerve entrapment may occur in the hip region and less commonly in the thigh, and clinical presentations are based upon the level of injury [3]. Sciatic neuropathy may result from conditions such as fibrous or muscular entrapment, vascular compression, scarring related to trauma (Figure 15) or radiation, tumors (Figure 16), and hypertrophic neuropathy [3, 17, 18]. Piriformis syndrome is a controversial diagnosis, often thought to be related to sciatic nerve compression or irritation related to the piriformis muscle. MRI can show variations in anatomy, muscle hypertrophy, as well as abnormal signal of the sciatic nerve [19]. MR imaging is not only a sensitive technique in identifying and characterizing the causative abnormalities but also can provide useful information for surgical planning [20].

### 4.2. Common Peroneal Nerve

**Figure 17:**
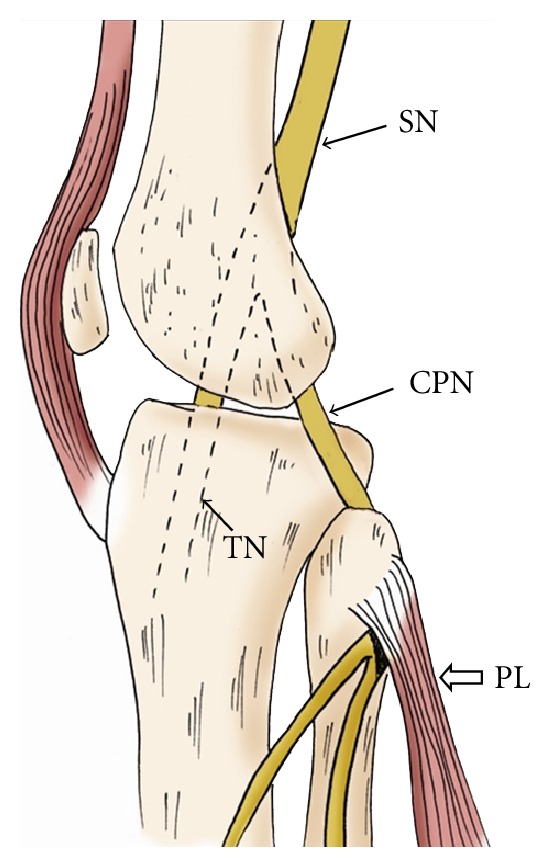
Sagittal oblique projection of the knee illustrates the common peroneal nerve (CPN) arising from the sciatic nerve (SN) at the level of popliteal fossa. It travels around the fibular head deep to the origin of the peroneus longus muscle (PL). TN: tibial nerve.

The common peroneal nerve arises from the sciatic nerve at the level of popliteal fossa. It travels distally and laterally posterior to the short head of the biceps femoris muscle, and lateral and superficial to the lateral head of the gastrocnemius muscle. It then passes around the fibular head laterally entering the anterolateral aspect of the leg deep to the peroneus longus muscle (Figure 17), where the nerve splits into deep and superficial peroneal branches.

**Figure 18:**
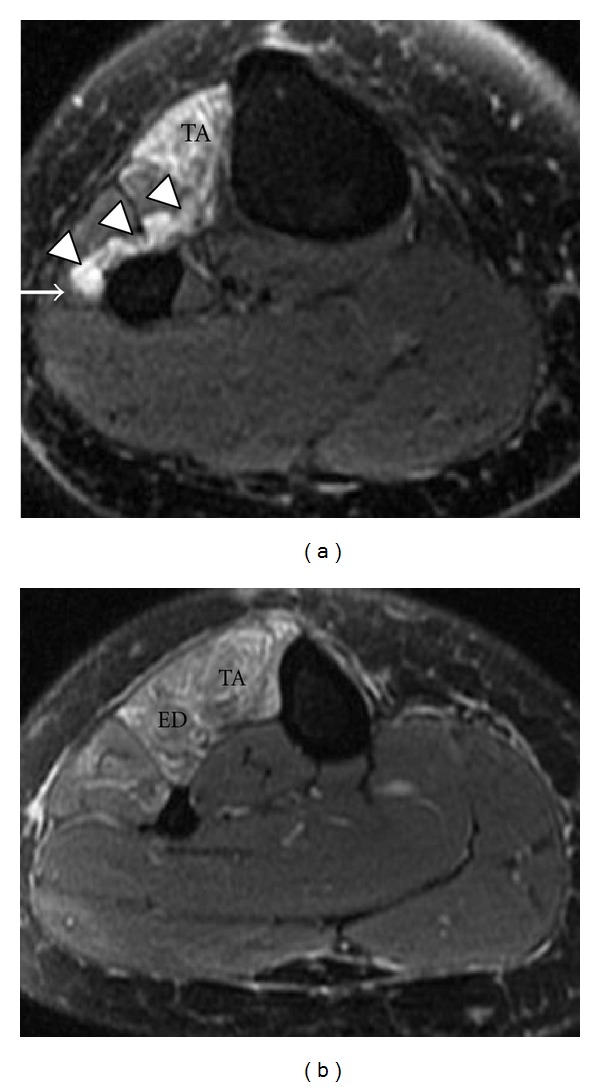
Common peroneal nerve entrapment secondary to a surgically proven intraneural ganglion cyst in a 44-year-old patient with a 6-month history of right foot drop. Axial T2-weighted fat-saturated images (a, b) reveal a multilobulated high T2 signal structure (arrowheads) compressing the adjacent common peroneal nerve (arrow). Associated patchy high signal in tibialis anterior (TA) and extensor digitorum longus (ED) muscles.

Nerve impingement of the common peroneal nerve may occur around the level of fibular head due to its superficial location, or as it travels deep to the origin of the peroneus longus muscle [17]. The etiologies of common peroneal neuropathy may include idiopathic mononeuritis, intrinsic and extrinsic space-occupying lesions including an intraneural ganglion cyst (Figure 18) [21], or traumatic injury of the nerve, especially related to proximal fibular fractures [22]. Clinically, patients may experience pain at the site of entrapment with foot drop and a slapping gait [17, 23].

 MR imaging is superior in depicting the location and cause of peroneal nerve compression and assessing the stage of the neuropathy, indicated by early muscle denervation or later changes such as atrophy.

### 4.3. Tibial Nerve

**Figure 19:**
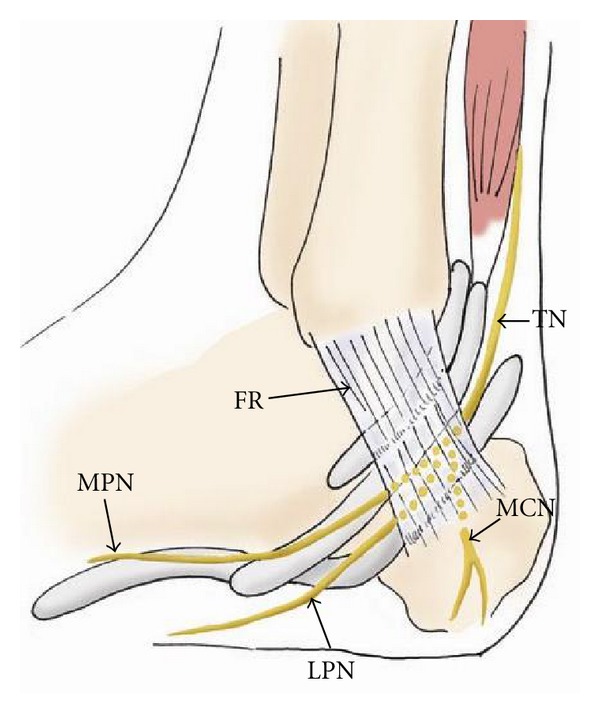
The drawing of the medial aspect of the ankle showing the course of the tibial nerve (TN) and its branches, the medial calcaneal nerve (MCN), and medial and lateral plantar nerves (MPN and LPN), passing through the tarsal tunnel. FR: flexor retinaculum.

After dividing from the sciatic nerve, the tibial nerve descends into the posterior compartment of the lower leg deep to the soleus, plantaris, and gastrocnemius muscles. It then crosses the ankle behind the medial malleolus, where it divides into its terminal branches, the medial calcaneal nerve, and medial and lateral plantar nerves. The name of the “posterior tibial nerve” is used as the tibial nerve reaches the ankle region [17]. The tarsal tunnel refers to a fibroosseous tunnel in the medial aspect of the ankle with the flexor retinaculum as the roof [17, 23]. The tunnel contains the flexor digitorum longus and flexor hallucis longus tendons, and the posterior tibial artery and veins, and the posterior tibial nerve and its branches (Figure 19).

**Figure 20:**
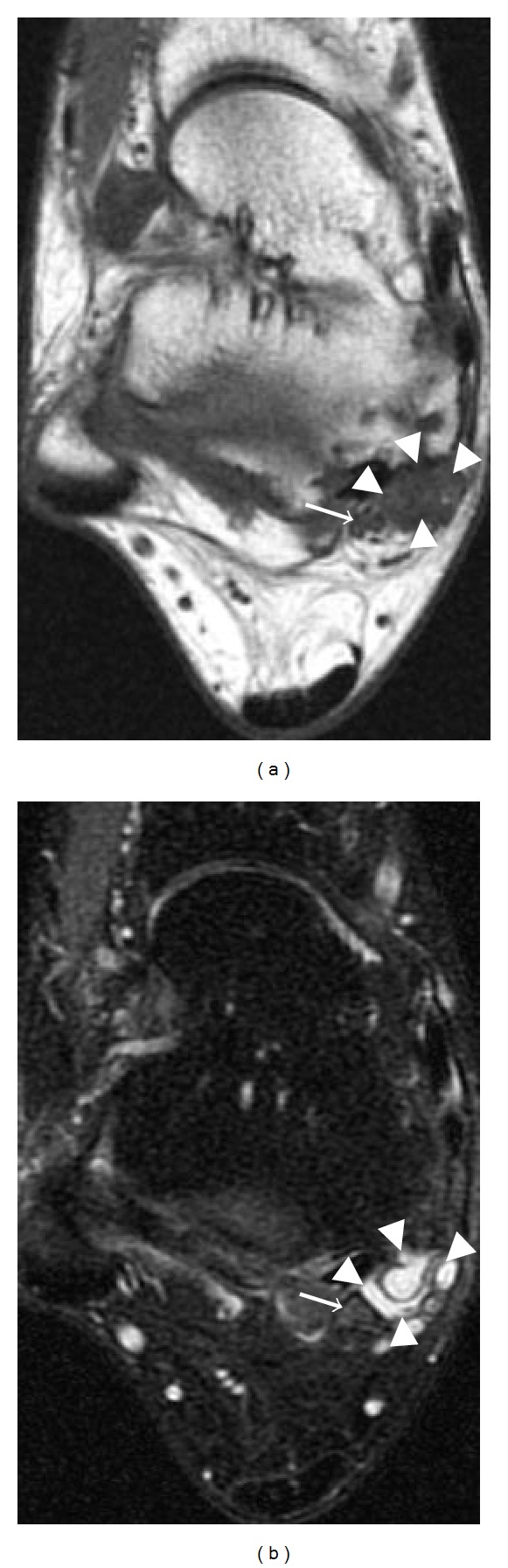
Tarsal tunnel syndrome caused by a ganglion cyst in a 32-year-old patient. Axial T1-weighted (a) and T2-weighted (b) fat-saturated images show a multilobulated cystic structure (arrowheads) within the right tarsal tunnel. Note the adjacent tibial nerve (arrows).

Tarsal tunnel syndrome is a well-known compression/entrapment neuropathy of the posterior tibial nerve. Common etiologies include posttraumatic fibrosis due to fracture, tenosynovitis, ganglion cysts (Figure 20), space-occupying lesions, and dilated or tortuous veins. Most patients with tarsal tunnel syndrome have burning pain and paresthesia along the plantar foot and toes. MR imaging is useful for localizing pathologies within the tarsal tunnel and depicting the lesion extent and relationship to the nerve and branches [24].

Compression of the proximal tibial nerve, the so-called soleal sling syndrome, is uncommon. It occurs when the proximal tibial nerve travels beneath the tendinous sling at the origin of the soleus muscle [25, 26]. The clinical presentation includes numbness, paresthesias in the sole of the foot, and posterior calf pain. MR imaging is useful in detecting increased T2 signal intensity of the nerve, as well as signal alteration in denervated gastrocnemius and soleus muscles [25].

### 4.4. Interdigital Nerve

The medial and lateral plantar nerves, which are terminal branches of the tibial nerve, divide into interdigital nerves at the level of metatarsal bases. The interdigital nerves pass deep to the transverse intermetatarsal ligament into a relatively small space between the metatarsal heads.

**Figure 21:**
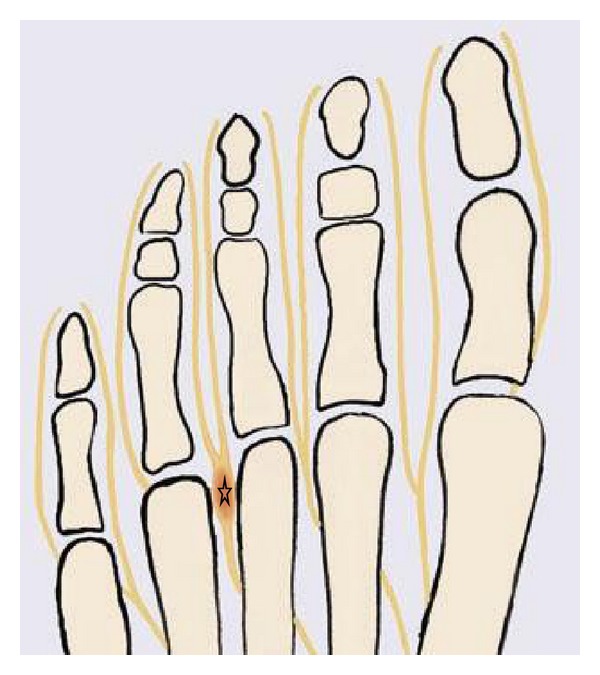
The drawing of the forefoot shows a Morton neuroma (star) at the site of the entrapment of the interdigital nerve between the third and fourth metatarsal heads.

**Figure 22:**
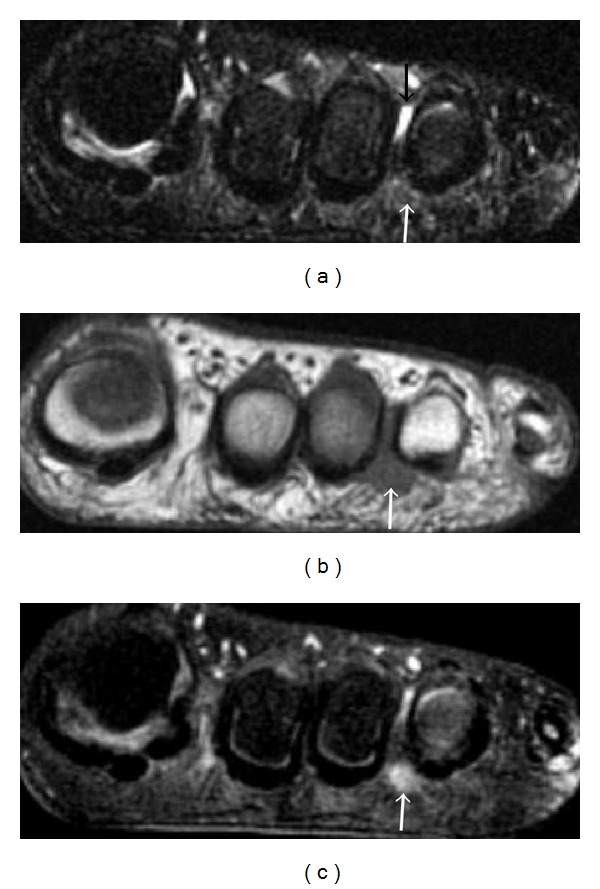
Morton neuroma in a 38-year-old patient. Coronal T2-weighted with fat-saturation (a), T1-weighted (b), and T1-weighted fat-saturated postcontrast (c) images identify an enhancing tear-drop-shaped soft tissue mass (white arrows) with intermediate signal on both T1- and T2-weighted images in the third intermetatarsal space. A small amount of fluid (black arrow) is noted within the intermetatarsal bursa dorsal to the neuroma in (a).

While repetitive mechanical stress with subsequent perineural fibrosis is the most commonly accepted cause of Morton neuroma, other possibilities include ischemia and compression of the nerve by an inflamed and enlarged intermetatarsal bursa [27]. Morton neuroma most frequently occurs in the second and third intermetatarsal spaces, often associated with an intermetatarsal bursa (Figure 21). The MRI appearance of the Morton neuroma is characteristic, typically manifested as an enhancing tear-drop-shaped soft tissue mass with intermediate signal on both T1- and T2-weighted images between the metatarsal heads (Figure 22). MR imaging provides very helpful information in localization and accurate size assessment of Morton neuromas.

## 5. Conclusion

Peripheral neuropathies may be underdiagnosed in patients with complicated clinical presentations. MR imaging provides valuable information in making a precise diagnosis and ready differentiation from other etiologies. Furthermore, it also helps make decisions for surgical planning. It is critical for radiologists to be familiar with the anatomy of the peripheral nerves and the range of pathology which may produce compressive/entrapment syndromes.
